# **IUCrJ** celebrates its first year of publication

**DOI:** 10.1107/S205225251402750X

**Published:** 2015-01-01

**Authors:** S. Samar Hasnain

**Affiliations:** a Max Perutz Professor of Molecular Biophysics, University of Liverpool, Crown Street, Liverpool, Merseyside L69 7ZB, United Kingdom, and Editor-in-chief of IUCr Journals

**Keywords:** crystallography, editorial, IUCrJ

## Abstract

**IUCrJ** is a fully open-access journal that publishes high-quality structural science papers. It has had a successful first year of publication.

To coincide with the International Year of Crystallography (IYCr2014), IUCr Journals launched the comprehensive open-access journal **IUCrJ** in January 2014. The journal has had an excellent first year, and has already started to establish itself within the wider scientific communities that use results obtained from diffraction methods. All the indications are good in terms of the journal making a strong impact in attracting high-quality science papers of wide scientific significance from these communities. First impressions from authors, readers, referees and commentators are very positive with a number of papers receiving high downloads in line with high-impact publications.

In addition to providing high-impact high-profile publication, **IUCrJ** also aims to provide fast publication for authors. Submissions undergo preliminary screening by a panel consisting of the five Main Editors (Ted Baker, Richard Catlow, Gautam Desiraju, Sine Larsen, John Spence) and the Editor-in-chief (Samar Hasnain), and this has helped to provide a rapid and efficient review process. Preliminary screening is generally complete within 72 hours, and any articles that do not meet the journal’s requirement for broad scientific significance are usually transferred, with the agreement of the authors, to one of our other journals. Such transfers are seamless and do not require any further work by the authors.

The six issues of **IUCrJ** published in 2014 have featured papers from a wide variety of areas including biology, chemistry, crystal engineering, materials, physics and FELs. The number of articles submitted to the journal in its first year was 130; this was well ahead of our target of 100 articles. A total of 72 papers have been published with an average turnaround time of 14 weeks. A number of papers have been highlighted *via* an in-depth commentary in a manner similar to other comprehensive journals such as *Nature* and *PNAS*.

**IUCrJ** has set out to become the natural home for reporting breakthroughs and ‘full’ science reports rather than simply reporting a structure or how it was determined. We welcome your impressions of the first year of **IUCrJ** and ideas of measures we should collectively take for its widest acceptance by the broadest possible community. We feel that we have made an excellent start and have attracted many high-quality submissions in all of the areas we cover. To maintain this confident start we encourage continued pro-active engagement from the whole structural community in improving the rate of submission of high-quality papers across the board. 

We want **IUCrJ** to become the leading journal for high-quality structure-based papers in the chemical and biological sciences. We remind these communities to consider **IUCrJ** as one of their first choice journals for their work that may have broader appeal. In addition, we will continue to work closely with physicists, material scientists, computational crystallographers and pioneering FEL scientists to ensure that **IUCrJ** is able to meet the expectations of these communities. 

As mentioned above, in 2014, we set ourselves a target of attracting 100 papers to celebrate IYCr2014 and the 100 years of success following the first Nobel prize related to crystallography, awarded in 1914 to Max von Laue. We are grateful to the community for helping us to exceeded this target by a significant margin. We are planning to publish 100 high-quality papers in 2015 in celebration of the centenary of W. H. and W. L. Bragg who won the Nobel prize in 1915 for the first use of X-rays to determine a crystal structure.

Throughout the year we plan to encourage a greater number of papers reporting advances in technologies and methods that underpin our structural science. In addition to advances associated with synchrotron sources and X-ray free-electron lasers, we look forward to reporting major advances that are taking place in neutron sources, methods and applications.

In 2015, we will be present at many major conferences including SRI 2015 (USA), XAFS 16 (Germany), IUBMB (Brazil), IUPAC (Korea), ACA (USA) and ECM (Croatia). Please come and visit the IUCr Journals stand, meet the Editors and get involved in making **IUCrJ** one of the mainstream comprehensive science journals. We look forward to seeing you there.

